# Exacerbation of Neonatal Hemolysis and Impaired Renal Iron Handling in Heme Oxygenase 1-Deficient Mice

**DOI:** 10.3390/ijms21207754

**Published:** 2020-10-20

**Authors:** Aleksandra Bednarz, Paweł Lipiński, Rafał R. Starzyński, Mateusz Tomczyk, Izabela Kraszewska, Sylwia Herman, Kacper Kowalski, Ewelina Gruca, Aneta Jończy, Rafał Mazgaj, Mateusz Szudzik, Zenon Rajfur, Zbigniew Baster, Alicja Józkowicz, Małgorzata Lenartowicz

**Affiliations:** 1 Department of Genetics and Evolution, Institute of Zoology and Biomedical Research, Jagiellonian University, Gronostajowa 9, 30-387 Kraków, Poland; a.j.bednarz@doctoral.uj.edu.pl (A.B.); sylwiaherman@op.pl (S.H.); kacper1.kowalski@student.uj.edu.pl (K.K.); ewelinagruca96@gmail.com (E.G.); 2 Department of Molecular Biology, Institute of Genetics and Animal Biotechnology, Polish Academy of Sciences, Jastrzębiec, 05-552 Magdalenka, Poland; p.lipinski@igbzpan.pl (P.L.); r.starzynski@igbzpan.pl (R.R.S.); a.jonczy@igbzpan.pl (A.J.); r.mazgaj@igbzpan.pl (R.M.); m.szudzik@igbzpan.pl (M.S.); 3 Department of Medical Biotechnology, Faculty of Biochemistry, Biophysics and Biotechnology, Jagiellonian University, Gronostajowa 7, 30-387 Kraków, Poland; tomczyk.mateusz@gmail.com (M.T.); izabela.kraszewska@doctoral.uj.edu.pl (I.K.); alicja.jozkowicz@uj.edu.pl (A.J.); 4 Department of Molecular and Interfacial Biophysics, Faculty of Physics, Astronomy and Applied Computer Science, Jagiellonian University, Łojasiewicza 11, 30-348 Kraków, Poland; zenon.rajfur@uj.edu.pl (Z.R.); zbigniew.baster@doctoral.uj.edu.pl (Z.B.)

**Keywords:** iron, heme, heme oxygenase 1, *Hmox1* gene, neonatal hemolysis, renal iron handling

## Abstract

In most mammals, neonatal intravascular hemolysis is a benign and moderate disorder that usually does not lead to anemia. During the neonatal period, kidneys play a key role in detoxification and recirculation of iron species released from red blood cells (RBC) and filtered out by glomeruli to the primary urine. Activity of heme oxygenase 1 (HO1), a heme-degrading enzyme localized in epithelial cells of proximal tubules, seems to be of critical importance for both processes. We show that, in HO1 knockout mouse newborns, hemolysis was prolonged despite a transient state and exacerbated, which led to temporal deterioration of RBC status. In neonates lacking HO1, functioning of renal molecular machinery responsible for iron reabsorption from the primary urine (megalin/cubilin complex) and its transfer to the blood (ferroportin) was either shifted in time or impaired, respectively. Those abnormalities resulted in iron loss from the body (excreted in urine) and in iron retention in the renal epithelium. We postulate that, as a consequence of these abnormalities, a tight systemic iron balance of HO1 knockout neonates may be temporarily affected.

## 1. Introduction

Heme oxygenase 1 (HO1, coded by the *Hmox1* gene) is an inducible, stress-responsive, multifunctional enzyme playing an important role in inflammation and iron homeostasis. It catabolizes free heme, an iron protoporphyrin (IX), into equimolar amounts of ferrous iron (Fe^2+^), carbon monoxide (CO), and biliverdin [1]. Heme is an essential protein cofactor and signaling molecule [2]; however, on the other hand, it can exert cytotoxic effects via lipid peroxidation, protein cross-linking, and DNA damage. Therefore, intracellular heme concentration in so-called labile heme pools, available for trafficking within the cell, should be strictly regulated [3]. HO1 by degrading heme contributes to heme homeostasis and to the protection against free heme-induced toxicity. This latter function requires the co-expression of H-ferritin, which, by sequestering ferrous iron released from the protoporphyrin IX ring, neutralizes their pro-oxidant effects [4]. Importantly, in macrophages, which ingest senescent red blood cells, hemoglobin-derived heme is rapidly degraded by means of the catabolic activity of HO1, and then iron extracted from heme is exported by ferroportin, a membrane-bound molecule, into the circulation. Iron is transported in the blood in the complex with transferrin to the bone marrow for sustaining physiological erythropoiesis [5]. This pathway of heme iron recycling represents the main physiological route of iron egress in the body and, therefore, HO1 is a key enzyme in systemic iron turnover.

In mice, HO1 deficiency results in serious perturbations in iron reutilization [6,7,8,9], which is directly associated with the inability of erythrophagocytosing macrophages to catabolize heme and their progressive loss during the lifespan [7,9]. HO1-deficient mice progressively developed intravascular hemolysis with age [6,7,8]. Hemolysis has also been diagnosed in two cases of human HO1 deficiency [10,11]. Hemolysis is a pathological condition, upon which a large amount of hemoglobin and/or heme is released into the plasma from damaged red blood cells. Together with haptoglobin and hemopexin (hemoglobin- and heme-binding plasmatic proteins, respectively), HO1 is as an effective protective agent against vascular injury caused by exogenous heme, reported in hemolytic disorders such as sickle-cell anemia [12].

The enhanced susceptibility of neonatal erythrocytes to increased oxidative stress is very likely the main reason of moderate intravascular hemolysis, which is a common, physiological condition in newborns. Interestingly, the course of neonatal hemolysis runs in parallel with developmentally impaired iron handling by liver macrophages [13]. Keeping all proportions, these phenomena are reminiscent of hemolytic events and macrophage dysfunction in HO1-deficient mice. We have recently demonstrated that, in mouse neonates showing clear symptoms of hemolysis, the kidney is the primary organ involved in heme uptake and catabolism and, thus, contributes to its detoxification, as well as to heme-derived iron recirculation from the primary urine to the blood [13]. Moreover, we have shown that, apart from heme and iron transporters, HO1 is exclusively expressed in the renal cortex and plays a pivotal role in these processes. Indeed, there is large evidence from human and animal studies that, in intravascular hemolytic disorders, kidneys and renal HO1 are involved in handling iron entering the circulation and then filtered out by glomeruli to the primary urine [14].

In this study, we explored the role of HO1 in renal iron homeostasis under conditions of neonatal hemolysis, using mice with the knockout of *Hmox1* gene (*Hmox1^-/-^*^)^. Because *Hmox1^−/−^* mice during the neonatal period were not characterized in terms of changes in systemic iron metabolism, we addressed this issue with a special emphasis on renal iron. Our results showed that, in *Hmox1^−/−^* neonates, hemolysis is extended and strongly aggravated compared to wild-type (WT) ones, which results in deepened hemolytic anemia. Similarly to WT mice, kidneys of *Hmox1^−/−^* animals take over the task of iron handling during neonatal hemolysis; however, their function due to the lack of HO1 activity is impaired. In consequence, renal iron recycling is inefficient, causing substantial loss of iron with urine.

## 2. Results

### 2.1. Increased Hemolysis and Decreased Red Blood Cell Status in Hmox1^−/−^ Mouse Neonates

**Figure 1:**
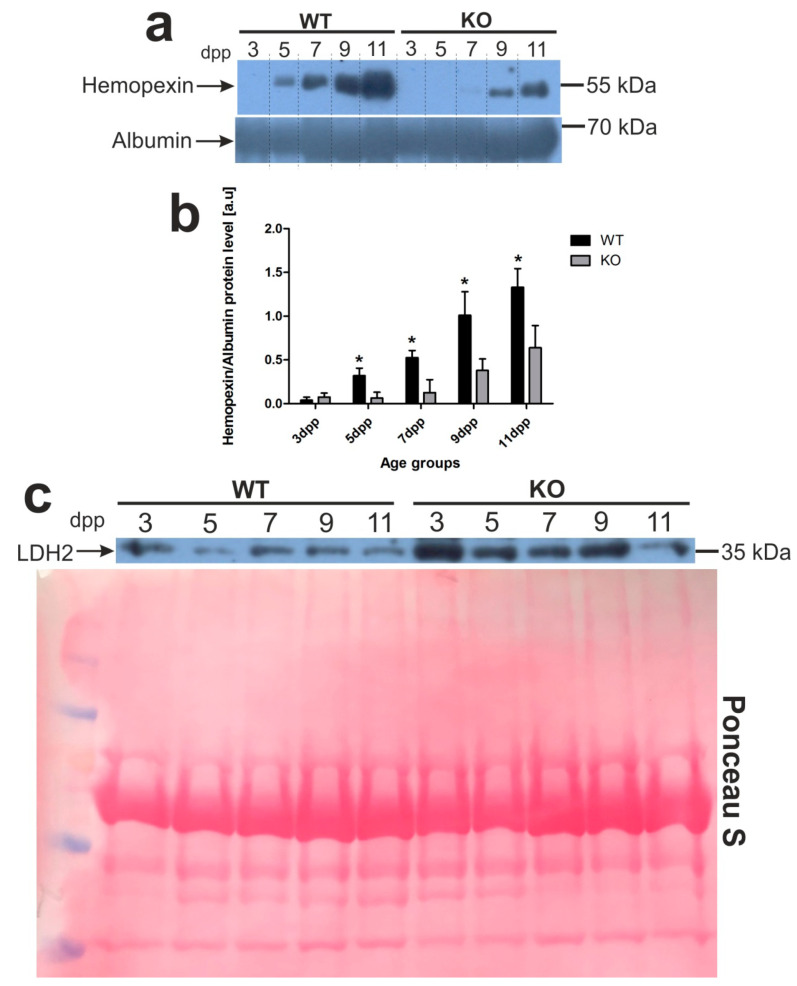
Hemopexin (Hx) and lactate dehydrogenase 2 (LDH2) proteins are markers of intravascular hemolysis in the blood of wild-type and *Hmox1*-deficient neonatal mice. (**a**) Plasma levels of hemopexin (Hx) were assessed by Western blotting. The blots were reprobed with polyclonal anti-albumin antibody as a loading control. Results come from three separate blots and relative levels of proteins are shown in the graph (**b**). Immunolabeled Hx and albumin control bands were quantified using a Molecular Imager and the relative levels of the test proteins (means ± SD, *n* = 3 per group) are plotted in arbitrary units. To compare differences in plasma Hx levels between wild-type (WT) and heme oxygenase 1 (HO1) knockout (KO) groups at each time point, we used Student *t*-tests for all datasets; * *p* < 0.05. (**c**) Lactate dehydrogenase 2 (LDH2) plasma levels in the serum from all age groups of wild-type and *Hmox1*^−/−^ mice analyzed by Western blotting (WB; representative blot, *n* = 3). WB PVDF membrane stained with Ponceau S was used as a protein loading control. dpp—days postpartum.

Recently, we determined a time-course of hemolysis in outbred mouse pups during the neonatal period [13]. Here, we confirmed those results using C57BL/6 × FVB mouse neonates. Furthermore, we showed that pups lacking heme oxygenase 1, a heme-degrading enzyme, develop much more severe and longer lasting hemolysis (Figure 1). To compare the time-course and intensity of hemolysis in wild-type (WT, *Hmox1^+/+^*) and *Hmox1*^−/−^ (HO1 KO) mice, we first determined blood plasma levels of hemopexin (Hx), the high-affinity heme-binding plasma protein, disappearance of which from the plasma is well recognized as a hallmark of hemolysis [15]. We found that Hx was barely detectable in the plasma of only 3 day old WT mice, whereas, in HO1 KO animals, it almost disappeared from the plasma between days 3 and 7 after birth (Figure 1a,b). Higher efficiency of recovery from hemolysis in WT mice was reflected by gradually higher Hx concentration in the plasma compared with *Hmox1*^−/−^ animals, starting from days 5 and 9 postpartum, respectively. Differences in the time-course and the intensity of hemolysis between *Hmox1^+/+^* and *Hmox1*^−/−^ mice were also confirmed by the results showing that the lactate dehydrogenase 2 (LDH2) plasma concentration (a marker for intravascular hemolysis) was maintained at higher levels in *Hmox1*^−/−^ mice throughout the entire analyzed neonatal period (Figure 1c). As a consequence of exacerbated hemolysis, 5 day old *Hmox1*^−/−^ pups showed significantly diminished values of most RBC indices such as hemoglobin concentration, hematocrit (HCT), mean corpuscular hemoglobin (MCH), and mean corpuscular volume (MCV) (Table 1). An increased MCH concentration (MCHC) value points to hemolysis as an etiological factor. Interestingly, in 11 day old knockouts, weakening hemolysis was accompanied by the overall recovery from the decline in RBC status, as RBC indices returned to normal values observed in age-matched WT mice.

### 2.2. Delay in Kidney Development and Renal Iron Loss in the Neonatal Hmox1^−/−^ Mice

**Figure 2:**
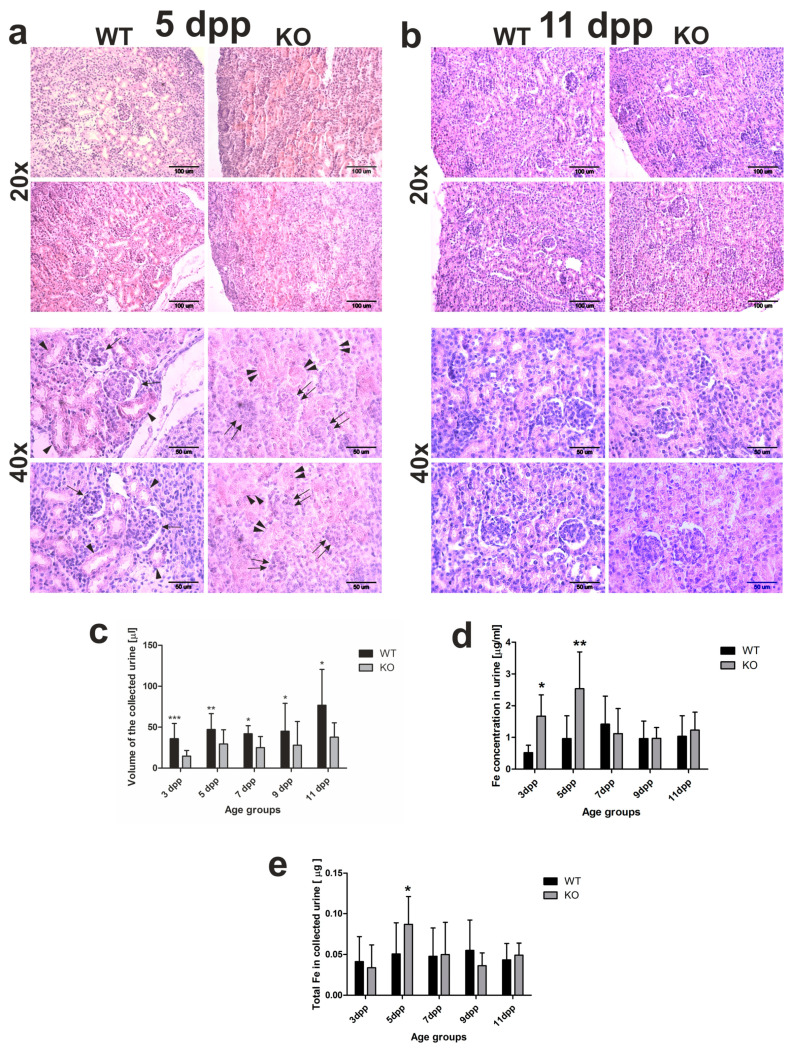
Kidney structure and urine analyses. (**a**,**b**) Hematoxylin–eosin staining of kidney sections from 5 and 11 day old (WT) and heme oxygenase 1 (HO1) knockout (KO) neonates; 20× magnification (upper panels) or 40× magnification (bottom panels). Scale bars correspond to 100 µm in 20× magnification and 50 µm in 40× magnification. (**c**) Iron concentration measured by AAS (atomic absorption spectroscopy) in urine samples of the WT and KO neonates from all age groups: *n* = 6 for WT 3 dpp, *n* = 10 for WT 5 dpp, *n* = 10 for WT 7 dpp, *n* = 5 for WT 9 dpp, *n* = 6 for WT 11 dpp, *n* = 5 for KO 3 dpp, *n* = 9 for KO 5 dpp, *n* = 5 for KO 7 dpp, *n* = 5 for KO 9 dpp, and *n* = 5 for KO 11 dpp. To compare differences in urine iron concentration between WT and KO groups at each time point, Student *t*-tests were used for all datasets. (**d**) Urine volume collected from urinary bladder of mice after dissection: *n* = 16 for WT 3 dpp, *n* = 19 for WT 5 dpp, *n* = 8 for WT 7 dpp, *n* = 12 for WT 9 dpp, *n* = 14 for WT 11 dpp, *n* = 14 for KO 3 dpp, *n* = 18 for KO 5 dpp, *n* = 7 for KO 7 dpp, *n* = 9 for KO 9 dpp, and *n* = 9 for KO 11 dpp. To compare differences in collected urine volumes between WT and *Hmox1* knockout (KO) groups at each time point, Student *t*-tests were used in 3 dpp, 5 dpp, 7 dpp, and 9 dpp age groups (where data distribution was normal) and a Mann–Whitney U test was used in the 11 dpp age group (where data distribution was not normal). (**e**) Iron content in urine collected from urinary bladder of mice after dissection: *n* = 10 for WT 3 dpp, *n* = 12 for WT 5 dpp, *n* = 9 for WT 7 dpp, *n* = 5 for WT 9 dpp, *n* = 6 for WT 11 dpp, *n* = 5 for KO 3 dpp, *n* = 9 for KO 5 dpp, *n* = 5 for KO 7 dpp, *n* = 5 for KO 9 dpp, and *n* = 5 for KO 11 dpp. To compare differences in total urine iron content between WT and *Hmox1* KO groups at each time point, we used Student *t*-tests in all age groups. Values are expressed as the means ± SD; * *p* < 0.05, ** *p* < 0.01, *** *p* < 0.001. dpp—days postpartum.

The kidneys are the main site of hemoglobin clearance and degradation under conditions of severe hemolysis [16,17,18]. The structural and functional maturity of the kidneys is indispensable for this process. In mice, in the first week of the postnatal life, nephrogenesis is still in progress, and approximately half of all nephrons is formed over days 2–4 after birth [19,20]. To see whether the process of nephrogenesis runs correctly in *Hmox1*^−/−^ neonates, we performed histological analysis of the kidneys from 5 day old mice. In the kidneys of the 5 day old WT mice, we observed renal corpuscles, with clearly distinguished renal glomeruli and formed Bowman’s space, located in the renal cortex among renal tubules (Figure 2a, arrows). However, in the renal cortex of 5 day old *Hmox1*^−/−^ mice renal corpuscles with developed Bowman’s space were barely detectable, although we were able to identify developed glomeruli (Figure 2a, double arrows). Moreover, we noticed that, in the kidneys of *Hmox1*^−/−^ mice, renal tubules were poorly developed in comparison with this part of the nephron in the kidneys of WT controls (Figure 2a indicated by double arrowheads and arrowheads, respectively). All these data indicate that the process of nephrogenesis is delayed in *Hmox1*^−/−^ pups. Interestingly, in 11 day old mice, the histological picture of the kidneys was very similar in both WT and *Hmox1*^−/−^ animals (Figure 2b).

To see whether differences in the histological structure of the kidneys directly translate into differences in kidney functioning, we performed a comparative analysis of urine volume in WT and *Hmox1*^−/−^ neonates. The obtained results indicate that, in neonates from all investigated age groups, the urine volume was significantly higher in wild-type mice than in *Hmox1*^−/−^ animals (Figure 2c). This suggests that the glomerular filtration is affected in *Hmox1*^−/−^ neonates. We also checked the total iron content in the urine of 3–11 day old mice of both genotypes. Atomic absorption spectroscopy (AAS) analysis revealed that, in the urine of 3 and 5 day old *Hmox1*^−/−^ neonates, iron concentration was significantly higher than in WT controls (Figure 2d,e). Interestingly, there were no statistically significant differences in iron content in urine in the 7–11 day old WT and *Hmox1*^−/−^ mice (Figure 2d,e).

### 2.3. Temporal Shift in the Increased Expression of Megalin-Cubilin Protein Complex in the Renal Proximal Tubules of Hmox1^−/−^ Neonates

**Figure 3:**
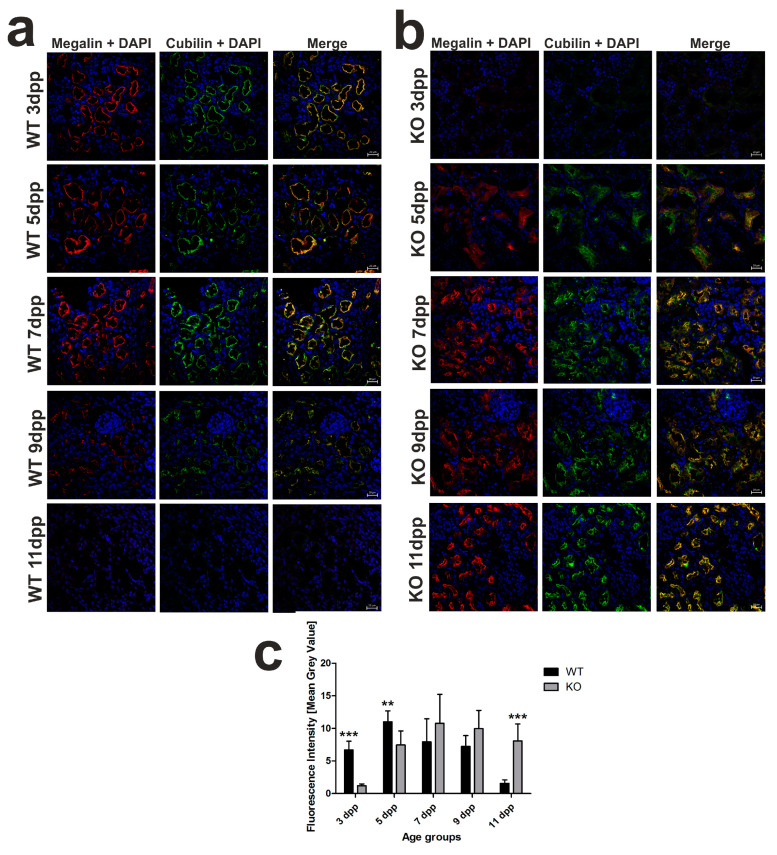
Localization of megalin–cubilin complex in renal proximal tubules in wild-type (WT) and heme oxygenase 1 (HO1) knockout (KO) neonatal mice. (**a**) Immunofluorescence (IF) staining of megalin (red channel) and cubilin (green channel) in the kidneys of the 3, 5, 7, 9, and 11 day old WT mice, analyzed by confocal microscopy. Both transporters have apical localization in the epithelial cells of cortical renal tubules. Cell nuclei were counterstained with DAPI (blue). Scale bars correspond to 20 μm. (**b**) IF staining of megalin (red channel) and cubilin (green channel) in the kidneys of the 3, 5, 7, 9, and 11 day old KO mice, analyzed by confocal microscopy. Both transporters are absent from epithelial cells of cortical renal tubules in 3 day old knockout mice; in 5 day old mice, they exhibit cytoplasmic localization, and, starting from day 7, both transporters have apical localization in the cells. Cell nuclei were counterstained with DAPI (blue). Scale bars correspond to 20 μm. (**c**) Quantitative analysis of co-localized fluorescent signal from megalin–cubilin complex in renal cortical tubules. The mean fluorescence signal associated with megalin and cubilin was measured on the whole renal cortex by ImageJ analysis and quantified manually as a mean gray value; the intensities (means ± SD) are plotted in arbitrary units (AU); *n* = 10 for WT 3 dpp, *n* = 9 for WT 5 dpp, *n* = 11 for WT 7 dpp, *n* = 8 for WT 9 dpp, *n* = 13 for WT 11 dpp, *n* = 6 for KO 3 dpp, *n* = 8 for KO 5 dpp, *n* = 14 for KO 7 dpp, *n* = 5 for KO 9 dpp, and *n* = 9 for KO 11 dpp. To compare differences in mean gray value (fluorescence intensity) between WT and *Hmox1* KO groups at each time point, Student *t*-tests were used in 3 dpp and 11 dpp groups, and Mann–Whitney U tests were used in 5 dpp, 7 dpp, and 9 dpp groups. ** *p* < 0.01, *** *p* < 0.001. dpp—days postpartum.

We previously showed that, in the kidneys of WT mouse neonates, the expression of megalin and cubilin, involved in hemoglobin clearance in the renal proximal tubules [16], was transiently (between days 3 and 7 after birth) increased in the apical membrane of the epithelial cells of the renal proximal tubules [13]. Here, immunofluorescent (IF) co-localization studies of megalin and cubilin showed that the increased megalin–cubilin IF signal in the apical part of the cortical renal tubules in *Hmox1*^−/−^ neonates was shifted in time compared to control animals and fell on days 7–11 postpartum (Figure 3a,b). In contrast to 3 day old WT neonates, we did not detect any megalin–cubilin IF signal in the kidney of *Hmox1*^−/−^ age-mates (Figure 3b). Interestingly, in 5 day old knockouts, we observed megalin–cubilin IF signals pointing to their intracellular localization in epithelial cells of the cortical renal tubules (Figure 3b). ImageJ analysis of the intensity of megalin/cubilin complex IF signals in the kidney of mice of both genotypes presented in Figure 3c revealed that the intensity of the IF signal for co-localization of megalin and cubilin (megalin/cubilin complex) was significantly higher in 3 and 5 day old WT than in KO neonates. These results indicate that, in the kidneys of the 3 and 5 day old *Hmox1*^−/−^ mice, reabsorption of hemoglobin from the primary urine was less effective than in wild-type neonates.

### 2.4. Unaffected Expression of Heme-Responsive Gene 1 (HRG1), A Heme Importer, in the Kidneys of Hmox1^−/−^ Neonates

**Figure 4:**
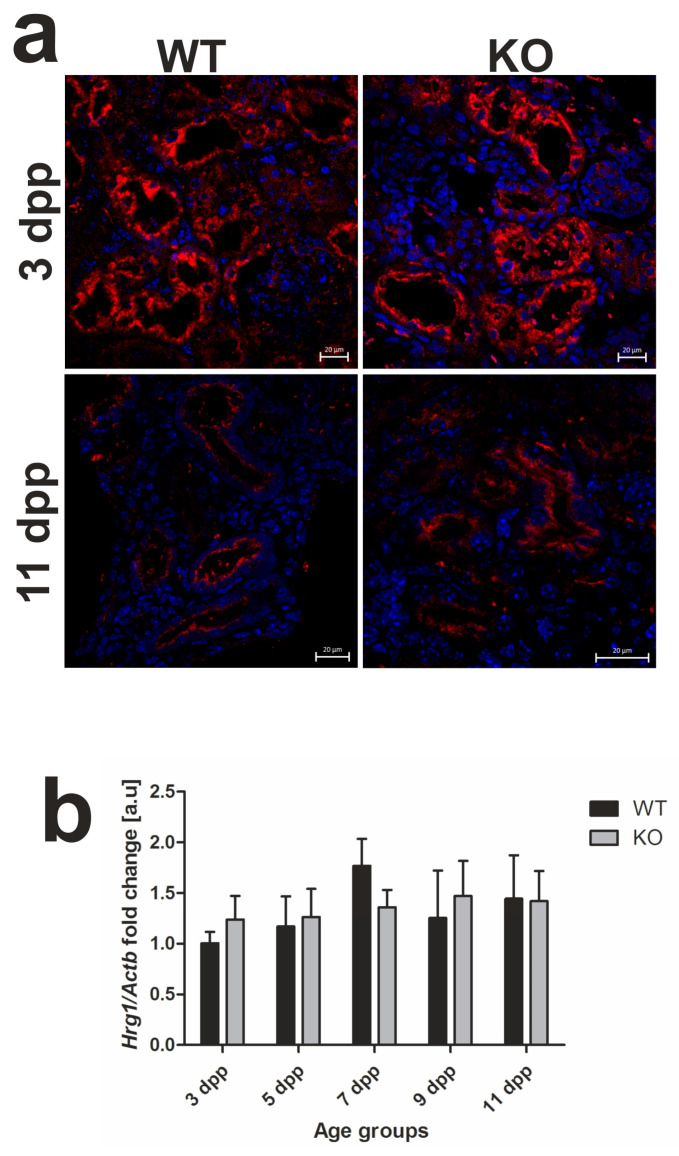

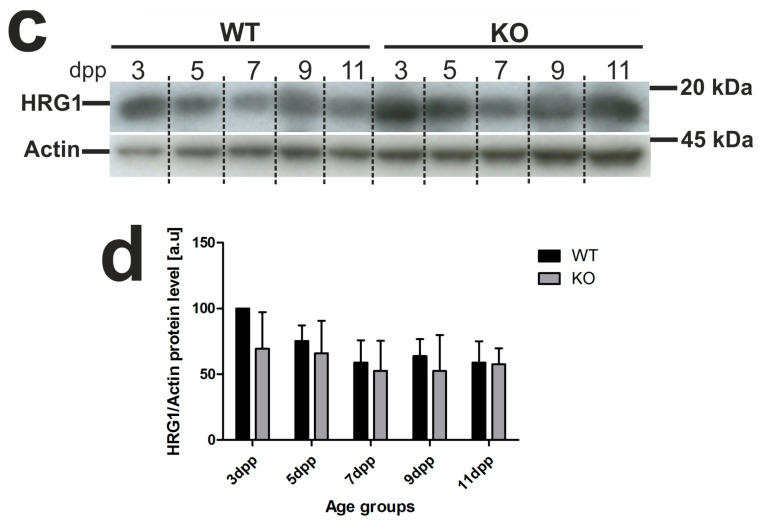
Heme transporter heme-responsive gene 1 (HRG1) in renal tissue. (**a**) Immunofluorescence (IF) staining of HRG1 (red channel) in the kidneys of 3 and 11 day old wild-type (WT) and heme oxygenase 1 (HO1) KO mice, analyzed by confocal microscopy. Cell nuclei were counterstained with DAPI (blue). Scale bars correspond to 20 μm. (**b**) RT-qPCR analysis of HRG1 (*Slc48a1*) messenger RNA (mRNA) levels in the kidneys of WT and KO mouse neonates. The graph presents relative HRG1 transcript levels in arbitrary units (means ± SD, *n* = 5 per group; Student *t*-test, NS). (**c**) HRG1 protein in kidneys assessed by Western blotting. The blots were reprobed with polyclonal anti-actin antibody as a sample loading control. Results come from four separate blots and relative levels of proteins are shown in the graph (**d**). Immunolabeled HRG1 and actin control bands were quantified using a Molecular Imager, and the relative levels of the tested proteins are plotted in arbitrary units (means ± SD, *n* = 4 per group; Student *t*-test, NS). dpp—days postpartum, NS—not significant.

In our previous study [13], we localized the HRG1 protein in apical/subapical regions of epithelial cells of the renal cortex in WT neonates, which suggested its participation in the uptake of free heme from primary urine under conditions of neonatal hemolysis. Here, we confirmed this localization in the kidneys of both WT and *Hmox1*^−/−^ mice (Figure 4a). Importantly, we show that, despite the enhanced hemolysis in *Hmox1*^−/−^ neonates, expression of the *Slc48a1* gene (encoding HRG1 protein) at both messenger RNA (mRNA; Figure 4b) and protein (Figure 4c,d) levels remains unaltered compared to WT mice throughout the entire experimental period.

### 2.5. Heme Oxygenase 1 (HO1) Deficiency Is Not Compensated for by the Increased Heme Oxygenase 2 (HO2) Expression in the Kidneys of Hmox1^−/−^ Mice

**Figure 5:**
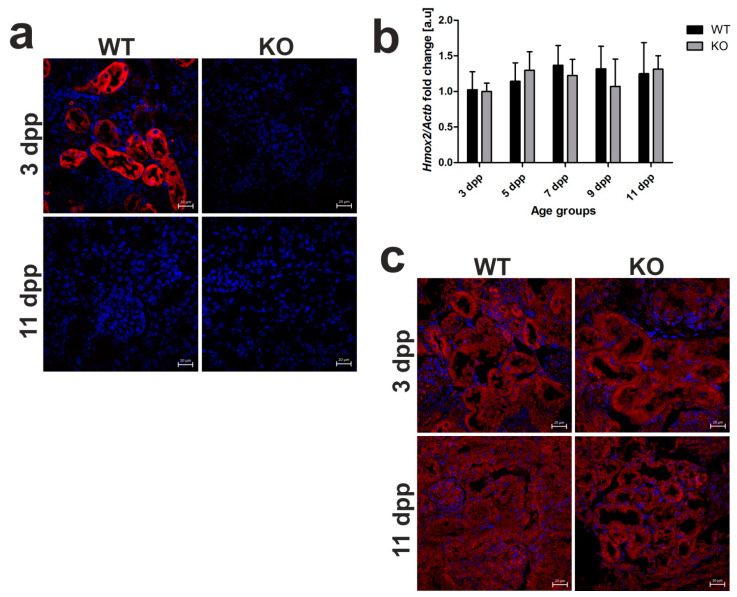
Heme oxygenase 1 (HO1) and heme oxygenase 2 (HO2) expression in the kidneys of wild-type and *Hmox1*-deficient neonatal mice. (**a**) Immunofluorescence (IF) staining of HO1 (red channel) in the kidneys of 3 and 11 day old wild-type (WT) and *Hmox1* knockout (KO) neonatal mice, analyzed by confocal microscopy. Cell nuclei were counterstained with DAPI (blue). Scale bars correspond to 20 μm. (**b**) RT-qPCR analysis of HO2 mRNA levels in the kidneys of WT and KO mouse neonates ranging from 3–11 days old. The graph presents relative HO2 transcript levels in arbitrary units (means ± SD, *n* = 5 per group; Student *t*-test, NS). (**c**) IF staining of HO2 (red channel) in the kidneys of 3 and 11 day old WT and KO neonatal mice, analyzed by confocal microscopy. Cell nuclei were counterstained with DAPI (blue). Scale bars correspond to 20 μm. dpp—days postpartum, NS—not significant.

In view of our previous results, HO1 located in the epithelial cells of proximal tubules is mainly responsible for the breakdown of heme taken up from the primary urine during neonatal hemolysis in outbred mice [13]. Here, we confirmed HO1 renal localization in C57BL/6 × FVB mouse neonates and showed no HO1 IF signal in *Hmox1*^−/−^ animals (Figure 5a). To investigate the possible compensative contribution of HO2 to heme degradation in the absence of HO1, we assessed the expression of *Hmox2* gene at the mRNA level (Figure 5b) and localization of HO2 protein (Figure 5c) in the kidneys of neonates of both genotypes. First, IF detection of HO2 in renal sections showed clearly that this protein is localized in the epithelial cells of the renal tubules in 3 and 11 day old HO1 knockout mice (Figure 5c). Consistent with the recognized role of *Hmox2* as a housekeeping gene, expressed at relatively constant rates independently of pathophysiological conditions, HO2 mRNA levels in the kidneys of *Hmox1*^−/−^ neonates did not differ significantly compared to control mice and were relatively stable throughout the entire neonatal period (Figure 5b). Our results suggest that, under conditions of exacerbated hemolysis in *Hmox1*^−/−^ neonates, the lack of HO1 activity is not compensated for by the increased expression of HO2 enzyme in the kidney.

### 2.6. Residual Ferroportin (Fpn) Expression and Increased Iron Status in the Kidneys of Hmox1^−/−^ Neonates

**Figure 6:**
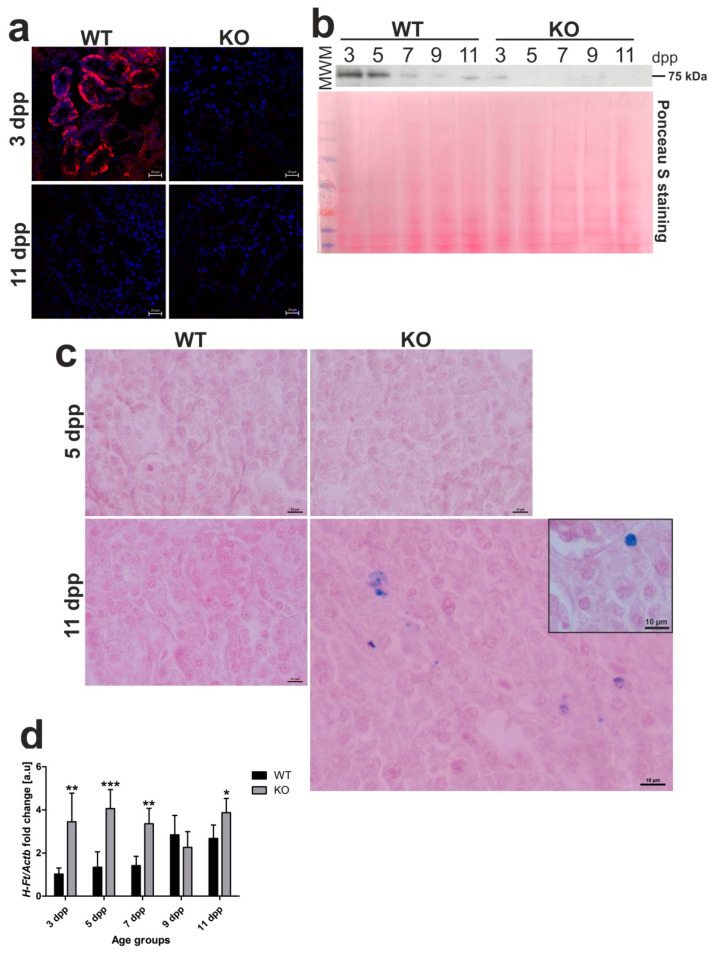
Ferroportin (Fpn) protein levels and localization, iron deposition, and H-ferritin (HFt) gene expression in kidneys of wild-type (WT) and heme oxygenase 1 (HO1) knockout (KO) mouse neonates. (**a**) Immunofluorescence (IF) staining of Fpn (red channel) in the kidneys of 3 and 11 day old (WT) and KO neonatal mice, analyzed by confocal microscopy. Cell nuclei were counterstained with DAPI (blue). Scale bars correspond to 20 μm. (**b**) Fpn protein levels in the kidneys of WT and KO mice from all age groups analyzed by Western blotting. WB PVDF membrane stained with Ponceau S was used as a protein loading control. (**c**) Histochemical analysis of iron loading in the kidneys of 5 and 11 day old WT and KO mouse neonates by Perl’s Prussian Blue staining. Iron deposits were detected in renal cortex of 11 day old KO mice (visible as blue deposits), whereas, in kidneys from WT mice of the same age and 5 day old mice of both genotypes, iron deposits were absent. Renal tissue was counterstained with pararosaniline (pink) and the scale bar corresponds to 10 µm. (**d**) RT-qPCR analysis of *H-Ft* mRNA levels in the kidneys of WT and KO mouse neonates ranging from 3–11 days old. The graph presents relative *H-Ft* transcript levels in arbitrary units (means ± SD, *n* = 5 per group). We used Student *t*-tests for all datasets; * *p* < 0.05, ** *p* < 0.01, *** *p* < 0.001. dpp—days postpartum, MWM—molecular weight marker.

It is well established that Fpn, the sole identified cell iron exporter [21], is localized to the basolateral membrane of the epithelial cells of proximal renal tubules where it functions as a protein releasing iron accumulated in the kidney into the circulation [8,22,23,24]. We recently showed that, under conditions of neonatal moderate hemolysis, expression of Fpn protein progressively declined in renal proximal tubules, and this decrease matched gradual disappearance of hemolysis in WT mouse neonates between days 3 and 11 after birth [13]. To check localization and expression of Fpn in *Hmox1*^−/−^ neonates, we performed immunofluorescent (IF) detection of Fpn in transverse renal sections of 3 and 11 day old mice of both genotypes (Figure 6a), as well as time-course Western blot analysis of Fpn levels in renal membrane extracts obtained from neonates from 3 to 11 days of age (Figure 6b). Microscopic analysis of IF staining indicated typical localization of Fpn in the basal membrane of epithelial cells of proximal renal tubules only in 3 day old WT neonates and disappearance of the IF Fpn signal in 11 day old mice, as reported recently [13]. In contrast, we did not detect an IF Fpn signal in both 3 and 11 day old *Hmox1*^−/−^ neonates. The results of Western blotting showed a gradual decrease in Fpn protein level in WT neonates, which contrasted with barely detectable Fpn levels in *Hmox1*^−/−^ animals throughout the entire neonatal period. There is a large body of evidence that decreased Fpn protein levels on the cellular membrane leads to excessive intracellular iron content [21]. Therefore, we examined iron accumulation in the kidney of 3 and 11 day old *Hmox1^+/+^* and *Hmox1*^−/−^ neonates via histochemical analysis, i.e., by staining the renal sections with Perls’ Prussian blue. We found blue iron deposits in sections across the renal cortex of the kidney of 11 day old *Hmox1*^−/−^ knockout neonates (Figure 6c). Iron accumulation in the kidneys of the *Hmox1*^−/−^ mice also led to increased expression of the H-ferritin (*H-Ft*) gene (Figure 6d). On the other hand, there was no evidence of iron loading in the kidneys of 11 day old WT neonates. On the basis of our results, it seems that drastically downregulated Fpn in epithelial cells of proximal tubules of *Hmox1*^−/−^ neonates is responsible for impaired recirculation and detoxification of iron accumulated in these cells.

### 2.7. Increased Hepcidin Expression in the Liver of 3 Day Old Hmox1^−/−^ Neonates

**Figure 7:**
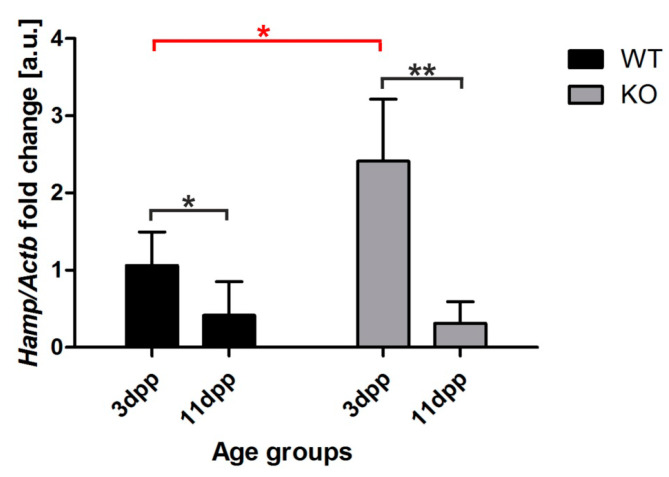
Hepcidin (*Hamp)* expression in the liver of wild-type (WT) and heme oxygenase 1 (HO1) knockout (KO) mice. RT-qPCR analysis of hepcidin mRNA levels in the livers of WT and KO 3 and 11 day old mouse neonates. The graph presents relative hepcidin transcript levels in arbitrary units (means ± SD, *n* = 5 per group). To compare differences, we used Student *t*-test; * *p* < 0.05, ** *p* < 0.01. dpp—days postpartum.

Hepcidin is a small peptide hormone that orchestrates body iron fluxes, produced mainly by hepatocytes. Hepcidin binds to Fpn to induce its degradation, thus inhibiting iron release from exporting cells [21]. Its expression is controlled by iron levels (circulating and liver iron content), erythropoietic activity, and inflammatory cues [25]. To find out the reason why ferroportin protein is barely detectable in the kidney of *Hmox1*^−/−^ neonates, we examined hepcidin mRNA abundance in the liver of 3 and 11 day old mice, comparing the obtained results with hepcidin transcript levels in the livers of age-matched control animals (Figure 7). We observed a strong, significant increase in hepcidin mRNA level in 3 day old HO1 knockouts compared to controls in the same age (Figure 7). We also found that hepcidin mRNA expression in the liver of 11 day old neonates of both genotypes did not differ and remained very low (Figure 7). These results suggest that increased hepcidin plays a role in the negative regulation of renal Fpn in 3 day old *Hmox1*^−/−^ neonates.

## 3. Discussion

Dysregulation of systemic iron homeostasis in HO1 deficiency, reflected by defective iron recycling and redistribution of tissue iron, was intensively explored in a mouse model [6,7,8] and in 6- and 15 year old patients [10,11]. It is evident from animal studies that abnormalities associated with HO1 deficiency develop progressively with age [6,9,26]. To the best of our knowledge, the 10 week old *Hmox1*^−/−^ mice are so far the youngest animals examined toward iron pathology, diagnosed as having no symptoms observed in older individuals [6]. Here, we examined for the first time changes in iron metabolism in *Hmox1*^−/−^ mouse pups between days 3 and 11 after birth. The main premise for this investigation was the key role of renal HO1 in iron redistribution during neonatal physiological intravascular hemolysis in wild-type mice [13]. We found a strong aggravation and an extension in time of neonatal hemolysis in *Hmox1^–/–^* mice, indicated by hemolysis markers such as decreased hemopexin and increased lactate dehydrogenase levels in the blood plasma. Exacerbated hemolysis in *Hmox1*^−/−^ mice resulted in the deterioration of RBC status in 3 day old neonates at the borderline of anemia. This points to a crucial role of HO1 in preventing hemolysis and shows that hemolytic anemia reported previously only in adult *Hmox1*^−/−^ mice [6,7,8] also temporarily affects neonates.

Kidney is an organ highly adapted to reabsorb both non-heme and heme iron filtered in the glomerulus to the primary urine and to recirculate it across epithelial cells of proximal tubules into the blood stream [14]. Under intravascular hemolysis conditions, such as chronic hereditary and acquired hemolytic anemias, kidneys participate in the management of iron released from disrupted erythrocytes to the blood plasma [27,28,29,30]. It is clear that the structural and functional maturity of the kidneys is indispensable for this process. Meanwhile, histological analysis of the kidney of 5 day old HO1 KO mice revealed that the renal corpuscle, a structure within which filtration process takes place, composed of the glomerulus surrounded by double-walled epithelial Bowman’s capsule, is not fully formed. This observation strongly suggests that, in KO mice, renal development is delayed in comparison with WT animals. It is known that vasculogenesis and angiogenesis contribute to the formation of glomeruli in the developing kidney [19]. Keeping in mind that HO1 facilitates angiogenesis through modulating expression of angiogenic factors [31], it is tempting to propose that the lack of HO1 activity can lead to the delay in glomeruli formation. Decreased volume of urine collected from *Hmox1*^−/−^ mice throughout the experimental period, as well as increased iron concentration in the urine falling for days with the greatest intensity of hemolysis, is a probable consequence of the developmental retardation of the morphological development of kidneys and functional renal insufficiency. Most importantly, increased total amount of iron excreted with urine was recorded just after the peak of hemolysis (day 5 after birth). This urinary loss of iron might contribute to worsening of a fragile systemic iron balance in knockout neonates. Therefore, a major question arises as to the efficiency of molecular mechanisms of renal iron handling in *Hmox1*^−/−^ neonates. Recently, in a time-course study, we explored the role of megalin-dependent, cubilin-mediated endocytic transport of iron from the primary urine by proximal tubules of epithelial cells during the neonatal hemolysis in wild-type mice [13]. The multi-ligand hetero-dimeric receptor complex megalin/cubilin present at the apical membrane of the tubular epithelium is responsible for the uptake of both hemoglobin and transferrin-bound iron [32]. Our present results show that the full expression of the complex in the renal proximal tubules of pups lacking HO1 is delayed few days compared to controls, i.e., it appears only in 7 day old *Hmox1*^−/−^ animals, while, in age-matched controls, it is already starting to decay. This time-shifted megalin–cubilin expression does not overlap with the hemolysis peak and probably explains why, in the absence of this complex in 5 day old *Hmox1*^−/−^ mice, iron is not reabsorbed by the renal epithelial cells and is excreted from the organism with urine.

To further assess the potential of epithelial cells of the renal cortex to scavenge heme iron from the primary urine during intensified neonatal hemolysis in *Hmox1*^−/−^ mice, we examined the heme transporter, HRG1 protein, (identified in the intestine of *Caenorhabditis elegans* [33]), in the kidney at extreme time-points of hemolysis development, i.e., on days 3 and 11 after birth. Surprisingly, we found in animals of both genotypes a similarly high and low level of the HRG1 protein at the beginning and at the end of hemolysis, respectively. Likewise, HRG1 protein showed the same localization in the proximal renal tubules in *Hmox1*^−/−^ and *Hmox1^+/+^* mice. It seems, therefore, that, upon exacerbated hemolysis in *Hmox1*^−/−^ neonates, HRG1 anchored in the apical membrane of epithelial cell in proximal tubules [13] is not upregulated, probably because free heme, a natural HRG1 ligand, is not a dominant heme iron compound appearing in the primary urine.

We recently provided evidence that HO1 is one of the most important players in renal iron handling under conditions of neonatal hemolysis [13]. In the absence of HO1 activity, the question always arises as to the potential compensation of this deficiency by HO2, a constitutively expressed enzyme that is involved in heme breakdown under physiological conditions [34]. Interestingly, recent studies indicated that young mice compared with aged animals (showing impairment in the inducibility of HO1) succeeded in the induction of HO2 in the kidney in response to hemoglobin [35]. However, here, we showed that, in the kidney of 3 and 11 day old *Hmox1*^−/−^ and control neonates, HO2 expression and distribution in the renal cortex and core were similar. Accordingly, in bone marrow-derived macrophages from adult HO1 KO mice, the expression of HO2 was not increased above the level detected in age-matched controls [7]. Tissue loading with non-heme iron is a real hallmark of HO1 deficiency [6,7,8]. Consistent with this is the accumulation of non-heme iron deposits in the kidney of 11 day old *Hmox1*^−/−^ mice. The question as to how intracellular heme iron is converted to non-heme iron in the absence of HO1 has never been fully answered. Poss and Tonegawa have suggested, that apart from HO2, a major contributor to endogenous HO activity and, therefore, non-heme iron release from heme molecules under basal conditions, alternative nonspecific and less efficient enzymatic systems of heme degradation become functional [6].

On the basis of our results, we propose a scheme of renal iron handling in HO1 deficiency during the neonatal period. Under conditions of increased hemolysis in *Hmox1*^−/−^ mouse newborns, the reabsorption of iron (mainly in the form of hemoglobin) filtered in the glomerulus to the primary urine is impaired due to the absence of the megalin/cublin complex on the apical membrane of the epithelial cells of renal proximal tubules. Then, as this complex appears (in 7 day old mice), hemoglobin is taken up by the renal epithelium. However, in the absence of HO1, the process of intracellular iron release from hemoglobin-derived heme molecules is slow, and detectable ferritin (Ft) iron deposits emerge only in 11 day old mice. It is well established that H and L subunits forming the molecule of ferritin, an iron storage protein that can accommodate up to 4500 atoms of iron [36], are mainly upregulated by non-heme iron via the IRP/IRE (iron regulatory protein/iron responsive element) post-transcriptional system [37]. Although we do not exclude such adjustment occurring in the *Hmox1*^−/−^ renal epithelium, it is worth noting that the level of H-ferritin mRNA was strongly elevated in *Hmox1*^−/−^ mice throughout the entire neonatal period. The *H-Ft* gene is known to be highly inducible by several environmental signals, including cytokine stimulation, stress signals, and a disease state [38]. Interestingly, it was reported that transcriptional regulation of the *H-Ft* gene is mediated by heme [39]. This regulation may involve heme-dependent stabilization of Nrf2 transcription factor, which in turn was demonstrated to induce *H-Ft* gene in response to xenobiotics [40]. We, therefore, suggest that an elevated labile heme pool [3] maintained in epithelial cells of *Hmox1*^−/−^ mice is responsible for the increase in *H-Ft* mRNA level. Undoubtedly, one of the main causes of iron retention in renal epithelial cells of *Hmox1*^−/−^ mice is the decreased level of ferroportin (Fpn) protein, the only known mammalian exporter of non-heme iron [41]. While, in control animals, Fpn gradually decreases from day 3 to day 11 after birth, in *Hmox1*^−/−^ neonates, it is barely detectable throughout the neonatal period. Initially (day 3 after birth), the low Fpn level may be due the high hepcidin expression in the liver of *Hmox1*^−/−^ mice, consistent with functioning of the hepcidin–ferroportin regulatory axis [42], which seems to be an overriding molecular mechanism that controls Fpn protein level. A possible mechanism underlying upregulation of hepcidin may involve bone morphogenetic protein 6 (BMP6), a main activator of hepcidin [43], expressed predominantly in hepatic endothelial sinusoidal cells and modulated by iron fluctuations [44]. In this context, it is noteworthy that, of all sites in the body, the endothelium may be on the front line of exposure to heme and non-heme iron released from RBCs upon intravascular hemolysis [45]. This may impact endothelial iron-dependent toxicity but may also affect BMP6-depndent signaling.

In conclusion, to the best of our knowledge, findings of this study provide for the first time evidence of impaired iron handling in the kidney of *Hmox1*^−/−^ mice in response to exacerbated intravascular hemolysis during neonatal period. Dysregulation of iron-related genes in the kidneys results in the loss of iron from the body with urine secretion and in disturbed iron transfer from the epithelial cells of renal tubules into the blood circulation. Our results also indicate that the decreased RBC status of *Hmox1*^−/−^ neonates associated with intravascular hemolysis is a transient abnormality that bears no mechanistic connection to severe microcytic anemia observed in aged *Hmox1*^−/−^ mice [6,7,8], resulting from reduced viability of erythrophagocytosing macrophages [7].

## 4. Materials and Methods

### 4.1. Mice

Male and female mice (C57BL/6 × FVB) homozygous for the nonfunctional *Hmox1* allele (*Hmox1*^−/−^, HO1 knockout) and control mice (C57BL/6 × FVB, *Hmox1^+/+^*) were used in experiments. HO1 knockout mice were obtained by crossing heterozygous *Hmox1^+/^*^−^ females and *Hmox1*^−/−^ males. Genotyping using DNA isolated from mice tails was performed by PCR analysis. We examined 3, 5, 7, 9, and 11 day old mice with the day of birth counted as day 1. Up to the day of sacrifice, all pups remained with their mothers. All animals were bred in animal facility of the Faculty of Biochemistry, Biophysics, and Biotechnology, Jagiellonian University in Krakow, under specific pathogen-free (SPF) conditions. Mice were maintained in individually ventilated cages, under controlled environmental conditions (12 h light/dark cycle, at approximately 23 °C). All animal procedures were in accordance with Guide for the Care and Use of Laboratory Animals (Directive 2010/63/EU of European Parliament) and approved by the First Local Ethical Committee on Animal Testing at the Jagiellonian University in Krakow (permission number: 136/2015, approval code: ZI/49/2015, approval date: 26.05.2015).

### 4.2. Sample Collection

Neonatal mice were anesthetized with 5% (*v*/*v*) isoflurane in air and then sacrificed by decapitation. Blood samples were taken from neck veins using heparinized tips and kept in heparin-coated tubes on wet ice for further processing. For plasma retrieval, samples were centrifuged at 800× *g*, 4 °C, for 10 min, frozen in liquid nitrogen, and stored at −80 °C for further analyses. Urine samples were taken directly from the urinary bladder, and the volumes of samples were measured using automatic pipette. Samples were frozen in liquid nitrogen and stored at −80 °C. Kidneys and livers were frozen immediately after the dissection and kept at −80 °C for further molecular analyses. Tissue samples for immunofluorescence analysis were fixed in 4% paraformaldehyde in phosphate buffered saline (PBS). For the preparation of paraffin-embedded sections, kidneys were fixed in Bouin’s solution for 24 h.

### 4.3. Measurement of Red Blood Cell Indices

Blood for hematological analysis was collected in EDTA-coated tubes (Microvette 100, Sarstedt). The red blood cell count (RBC), hemoglobin (HGB), hematocrit (HCT), mean corpuscular volume (MCV), mean corpuscular hemoglobin (MCH), mean corpuscular hemoglobin concentration (MCHC), and red cell distribution width coefficient of variation (RDW-CV) were determined using an automated hematology analyzer (Abacus Junior Vet 5; Diatron, Budapest, Hungary).

### 4.4. Western Blot Analysis

To determine plasma hemopexin (Hpx) and lactate dehydrogenase 2 (LDH2) levels, 10 µL samples of 25-fold diluted mouse blood plasma were resolved by electrophoresis on 10% SDS-PAGE gels. Heme-regulated gene 1 (*Hrg1/Slc48a1*) protein was detected in 20 µg of kidney membrane extracts (prepared as described previously [8]) following electrophoresis on 16% SDS-PAGE gel. Ferroportin (Fpn) protein was detected in 40 µg of kidney membrane extracts (prepared as described previously [8]) following electrophoresis on 10% SDS-PAGE gels. Electroblotting of the resolved proteins onto a PVDF membrane (Millipore, Burlington, MA, USA), where blocking and incubation with primary antibodies were performed as described previously [13]. The primary and secondary antibodies used for immunoblotting experiments are described in Table S1 (Supplementary Materials). For the quantitative analysis of protein content, reactive band intensities were quantified relative to those of albumin (plasma samples) or actin (kidney samples) by densitometric analysis using Quantity One software (Bio-Rad, Hercules, CA, USA).

### 4.5. Hematoxylin–Eosin Staining and Histological Analysis of the Kidney Sections

Following fixation in Bouin’s solution for 24 h, kidneys were stored in 70% ethanol before further preparation. The tissue was embedded in paraffin and 7 μm cross-sections were cut with a microtome (Reichert-Jung, Nussloch, Germany). The sections were stained with hematoxylin and eosin. Slides were examined by light microscopy (Olympus, type CH2, Hamburg, Germany).

### 4.6. Measurement of Iron Content in Urine

The level of iron in urine samples was measured by atomic absorption spectrophotometry (AAS). First, 20 µL of urine was diluted in 2 mL of boiling Suprapur-grade nitric acid (Merck, Darmstadt, Germany). After cooling to room temperature, each sample was suspended in 2 mL of deionized water. Reference material samples were prepared in a similar manner. The iron concentration was measured using the graphite furnace AAS technique (AAnalyst 800, Perkin-Elmer, Waltham, MA, USA). Three samples of pure nitric acid were used as blanks. In addition, three samples of a standard reference material, Fe mg/kg 197.94 ± 0.65, were used for normalization of the obtained data.

### 4.7. Immunofluorescence (IF) Analysis and Confocal Microscopy of Kidney Sections

After laparotomy, mouse kidneys were immediately dissected and fixed in 4% paraformaldehyde (Sigma-Aldrich, Saint Louis, MO, USA) in phosphate-buffered saline (PBS) at 4 °C for 24 h. Following two 30 min washes in PBS, the tissues were successively soaked in 12.5% and 25% sucrose (Bioshop, Burlington, ON, Canada) for 2 h and 7 days, respectively, at 4 °C. Renal samples were then embedded in Cryomatrix medium (Thermo Scientific, Waltham, MA, USA), frozen in liquid nitrogen, and sectioned in 20 μm slices using a cryomicrotome (Shandon, UK). The sections were washed in PBS for 10 min and permeabilized by bathing in PBS/0.1% Triton X-100 (Sigma-Aldrich, Saint Louis, MO, USA) for 20 min. Nonspecific antibody binding was blocked by incubating the tissue sections in PBS/3% bovine serum albumin (BSA) (Bioshop, Burlington, ON, Canada) for 1.5 h. For protein detection, sections were incubated overnight at room temperature with primary antibodies diluted in PBS/3% BSA. As a negative control, some sections were incubated without primary antibody. The primary and fluorochrome-conjugated secondary antibodies used in IF analysis are described in Table S2 (Supplementary Materials). Next, the sections were washed for 5 × 6 min with PBS/0.1% Triton X-100 and incubated for 1.5 h with secondary antibody diluted in PBS/3% BSA at room temperature. Finally, sections were washed for 10 min in PBS and mounted in Fluoroshield medium with DAPI (Sigma-Aldrich, Saint Louis, MO, USA). The presence of megalin and cubilin proteins in renal tissues was determined by double immunofluorescence localization. In order to distinguish the different proteins in experiment, the secondary antibodies were conjugated with different fluorochromes: Cy3 for megalin and Alexa488 for cubilin. IF was analyzed with a Zeiss LSM 710 confocal microscope (Carl Zeiss, Oberkochen, Germany) using a 40× objective and Zeiss ZEN software (Carl Zeiss, Oberkochen, Germany).

### 4.8. ImageJ Analysis of Immunofluorescence (IF) Images

ImageJ analysis was performed to characterize immunofluorescent signal from megalin and cubilin. Samples were prepared and analyzed on confocal microscope as described previously [13]. ImageJ software (NIH, Bethesda, MD, USA) was used to measure mean fluorescence coming from both megalin and cubilin in the whole renal cortex on each photograph. The signal intensity was quantified to generate a mean gray value, i.e., the sum of gray values in the selected area divided by the number of pixels within that area.

### 4.9. Real-Time Quantitative PCR (RT-qPCR)

Total cellular RNA was extracted from livers and kidneys of experimental mice using an RNA isolation kit (Macherey-Nagel, Dueren, Germany). The RNA was treated with DNAse-I (Macherey-Nagel) and reverse-transcribed using a High-Capacity complementary DNA (cDNA) Reverse Transcription Kit (Applied Biosystems, Foster City, CA, USA). Specific *Hmox2*, *Hamp*, *Hrg1*, and *HFt* fragments were then PCR amplified from this cDNA using the specific primers (Table S3, Supplementary Materials). As a control, primers amplifying the housekeeping beta actin gene (*Actb*) were used. For RT-qPCR analysis, standard curves were generated using serial dilutions of cDNA to determine the amplification efficiency of each primer pair. To evaluate the relative expression level compared to *Actb*, the 2^−ΔΔct^ method was used. Real-time PCR was performed with the Power SybrGreen quantitative PCR kit (Applied Biosystems) using a StepOne thermocycler (Applied Biosystems, Foster City, CA, USA).

### 4.10. Prussian Blue Staining of Kidney

Non-heme iron staining of kidney samples was analyzed using Accustain Iron Deposition Kit (Sigma-Aldrich, Saint Louis, MO, USA). Kidneys were dissected, immediately fixed in Bouin’s solution for 24 h, and stored in 70% ethanol before further preparation. After dehydration, samples were embedded in paraffin and cut into 7 μm sections with a microtome (Reichert-Jung, Nussloch, Germany). After mounting on glass slides, sections were deparaffinized, incubated with working solution containing Perls’ Prussian Blue for 30 min, counterstained with pararosaniline solution for 2 min, and analyzed under standard light microscopy on Olympus CH2 and Nikon Eclipse E600 microscopes (Nikon Instruments, Amsterdam, The Netherlands).

### 4.11. Statistical Analysis

Datasets were analyzed for normal distribution using the Shapiro–Wilk test. Differences between wild-type and HO1 knockout mice in each age group were compared by parametric, two-tailed, Student’s *t*-test for data with normal distribution and nonparametric, Mann–Whitney U test for data where distribution was not normal. A *p*-value <0.05 was considered statistically significant. For statistical analyses and graph preparation, we used Graph Pad Prism 5 (GraphPad Software, San Diego, CA, USA) and STATISTICA 13 software (StatSoft, Krakow, Poland)

## Figures and Tables

**Table 1:** Red blood cell (RBC) indices of 5 and 11 day old C57BL × FvB wild genotype (WT) and heme oxygenase 1 (HO1) knockout (KO) mice. Values are expressed as the means ± SD, *n* = 13 for 5 day old WT mice, *n* = 13 for 5 day old KO mice, *n* = 8 for 11 day old wild-type mice, and *n* = 8 for 11 day old *Hmox1* knockout mice. To compare differences in each blood parameter between wild-type (WT) and *Hmox1* knockout (KO) groups at each time point, we used Student *t*-tests for datasets where distribution was normal and Mann–Whitney U tests where data distribution was not normal. Abbreviations: RBC—red blood cells, HGB—hemoglobin, HCT—hematocrit, MCV—mean corpuscular volume, MCH—mean corpuscular hemoglobin, MCHC—mean corpuscular hemoglobin concentration, RDW-CV—red cell distribution width coefficient of variation, ns—not significant; * *p* < 0.05, ** *p* < 0.01, *** *p* < 0.001.

Blood Parameters	5 Day Old Mice			11 Day Old Mice		
	WT	KO	*p*	WT	KO	*p*
RBC (M/µL)	3.6 ± 0.56	3.26 ± 0.46	ns	6.34 ± 0.51	6.03 ± 0.31	ns
HGB (g/dL)	9.11 ± 1.17	7.79 ± 1.02	**	11.15 ± 1.86	10.59 ± 0.49	ns
HCT (%)	30.48 ± 5.14	23.8 ± 3.16	***	41.01 ± 2.78	37.51 ± 1.99	*
MCV (fL)	84.49 ± 3.50	73.09 ± 4.38	***	64.63 ± 1.19	62.25 ± 2.12	*
MCH (pg)	25.39 ± 1.29	24.07 ± 1.55	*	17.5 ± 2.03	17.55 ± 0.53	ns
MCHC (g/dL)	30.02 ± 1.39	33.09 ± 1.55	***	27.06 ± 3.24	28.19 ± 0.49	ns
RDW-CV (%)	21.54 ± 1.77	23.62 ± 2.36	ns	15.36 ± 1.46	17.03 ± 0.87	*

## Supplementary Materials

The following are available online at https://www.mdpi.com/1422-0067/21/20/7754/s1, Table S1: Antibodies used in immunoblotting; Table S2: Antibodies used in immunofluorescence analysis; Table S3: Primers used in real-time PCR analysis.
