# Supplementary material for: Exacerbation of Neonatal Hemolysis and Impaired Renal Iron Handling in Heme Oxygenase 1-Deficient Mice
Source: Int J Mol Sci. 2020 Oct 20;21(20):7754. doi: 10.3390/ijms21207754 (PMC7589678; doi:10.3390/ijms21207754)
Supplement: Supplementary file 1 [file ijms-21-07754-s001.pdf]

Supplementary Materials

Table S1 - ***Antibodies Used in Immunoblotting***

Target protein	Primary Ab	Primary Ab dilution	Secondary Ab	Secondary Ab dilution
Hemopexin	Mouse polyclonal, kind gift from E. Tolosano, University of Turin, Italy	1:2000	Goat anti-mouse polyclonal, #A5278 (Sigma-Aldrich)	1:20 000
Lactate dehydrogenase 2 (LDH2)	Rabbit polyclonal, Proteintech, #14824-1-AP	1:1000	Goat anti-rabbit polyclonal, #A6154 (Sigma-Aldrich)	1:20 000
Albumin	Rabbit polyclonal, Santa Cruz Biotechnology, #SC-50536	1:1000	Goat anti-rabbit polyclonal, #A6154 (Sigma)	1:20 000
Slc48a1 (HRG1)	Rabbit polyclonal, Novus, #QC13349	1:500	Goat anti-rabbit polyclonal, #A6154 (Sigma-Aldrich)	1:20 000
Ferroportin (Fpn)	Rabbit polyclonal, Alpha Diagnostic International, #MTP11-A	1:1000	Goat anti-rabbit polyclonal, #A6154 (Sigma-Aldrich)	1:20 000
Actin	Goat polyclonal, Santa Cruz Biotechnology, #SC-1615	1:2000	Donkey anti-goat polyclonal, #SC-2020 (Santa Cruz Biotechnology)	1:20 000

Table S2 - ***Antibodies Used in Immunofluorescence Analysis***

Target protein	Primary Ab	Primary Ab dilution	Secondary Ab/Fluorochrome	Secondary Ab dilution
HO1	Rabbit polyclonal, Enzo Life Sciences, #ADI-OSA-150-F	1:250	Goat anti-rabbit conjugated with Cy3® fluorochrome (Jackson Immunoresearch)	1:500
HO2	Rabbit polyclonal, Enzo Life Sciences, #ADI-OSA-200	1:200	Goat anti-rabbit conjugated with Cy3® fluorochrome (Jackson Immunoresearch)	1:500
Fpn	Rabbit polyclonal, Alpha Diagnostic, #MTP11-A	1:250	Goat anti-rabbit conjugated with Cy3® fluorochrome (Jackson Immunoresearch)	1:500
Slc48a1 (HRG1)	Rabbit polyclonal, kind gift from Prof. I. Hamza, University of Maryland, USA	1:100	Goat anti-rabbit conjugated with Cy3® fluorochrome (Jackson Immunoresearch)	1:500
Megalin	Rabbit polyclonal Abcam #ab76969	1 : 250	Goat anti-rabbit conjugated with Cy3® fluorochrome (Jackson Immunoresearch)	1:500
Cubilin	Sheep polyclonal Novus #AF3700	1 : 100	Donkey anti-sheep conjugated with Alexa488® fluorochrome (Jackson Immunoresearch)	1:500

Table S3 - *Primers Used in Real Time PCR Analysis*

Target gene	Forward primer (5′→3′)	Reverse primer (5′→3′)
Actb	GGCCAACCGTGAAAAGATGACCCA	TACGACCAGAGGCATACAGGGACAG
Hmox2	AACAGGTCACTTGGGGTAGG	TGCTCAGCCAGACAAAGGTA
HFt	GCTGAATGCAATGGAGTGTG	CAGGGTGTGCTTGTCAAAGA
Hrg1	GACTCTGATGCTGGGTGATGG	CTTCGTGGGTGCTCTCTTCTC
Hamp	TGTCTCCTGCTTCTCCTCCT	CTCTGTAGTCTGTCTCATCTGTTG
